# Supplementary material for: The Essential Role of H2S-ABA Crosstalk in Maize Thermotolerance through the ROS-Scavenging System
Source: Int J Mol Sci. 2023 Jul 31;24(15):12264. doi: 10.3390/ijms241512264 (PMC10418723; doi:10.3390/ijms241512264)
Supplement: Supplementary file 1 [file ijms-24-12264-s001.zip › ijms-2519403-supplementary.pdf]

Table S1. Primers name, entry number, and primer sequence used in this paper

Gene	Accession Number	Primer sequence (5'—3')
ZmTUB	NM_001111988.	F: AGAACTGCGACTGCCTCCAAAGG<br>R: AGATGAGCAGGGTGCCCATTC
ZmLCD1	NM_001138259.	F: AAGTGTTGAGGAAGGACAAGAG<br>R: GGCATCTCTCAAGACCTCATAC
ZmOAS-TL	NM_001366967.	F: GGCAAGTACCTCAAGGAGAAA<br>R: CTACTCCGTTTCCAGTGATGAG
ZmZEP	NM_001305856	F: CCAGCGTAAATTTCGTGAGATG<br>R: TGCTGCTCTAGTCTGGTAATTC
ZmNCED1	XM_008646423	F: CGACTCCACCCTCTCTCTATAA<br>R: GCGTGATCGAGTGGGTTATT
ZmAAO	NM_001177021	F: TCAAGGTCTTGACGGACGTG<br>R: CCCGTACCATCTTCCTTCGG
ZmTUB	NM_001111988.	F: AGAACTGCGACTGCCTCCAAAGG<br>R: AGATGAGCAGGGTGCCCATTC
ZmCAT1	NM_001254879.2	F: GGGTCCAGACACCTGTTATTG<br>R: AGTTACCCTCTCTGGTGTAGAA
ZmSOD4	NM_001112234.2	F: CGTCACCAGCAGGCTAGAAT<br>R: AGCCAACAGTCCAACACAGT
ZmGR1	NM_001305818.1	F:CTCTCACGAGTTTGAAGAGTCTCGTGG<br>R:CCAGCGCAGCATCCGAATCTATAA
ZmAPX1	NM_001370758.1	F:GATCTTGTGGCTGCAGCATG<br>R:GGTGGACTCGAATTGCAGGA
ZmMDHAR	NM_001196274.1	F:AAGTGGTGGAGAGAAGCTATTG<br>R:CTAGTCAGAGTCTTGGTGGAAAG
ZmDHAR1	NM_001147572.1	F:ATCTCTGGTCACTCCTGTAGAA<br>R:CTCGGAACCATCACTAGCATC
